# A New Method of Applying the Rubber Dam

**Published:** 1875-05

**Authors:** S. F. Ham

**Affiliations:** Boston.


### ARTICLE XIV.

### *A New Method of Applying the Rubber Dam.*

BY S. F. HAM, D. M. D., BOSTON.

Having had great difficulty in applying the Rubber Dam by the usual method of fingers and ligatures, and in many cases failing after a half hour's effort, it occurred to me that possibly the object might be accomplished more readily, by bringing into requisition the clamp and forceps at once, making them do the work heretofore done so unsatisfactorily by the fingers, as follows:

Let the rubber be punctured in the place desired, over the end of an instrument, as described by some good fellow (I do not recall his name), not long since, in one of the Dental Journals of the day, thus securing an unbroken edge, which I regard as of the utmost importance; now stretch the rubber till the aperture admits freely the beaks of the clamp; with the forceps in position, fold the rubber about them closely, so as not to obstruct the vision, and apply to the tooth in the ordinary manner.

Apply the Cogswell holder, and with a small right-angle burnisher, or round, smooth instrument, slip the rubber below the clamp on either side, and the work is done. There is no need whatever for ligatures of any sort in a large majority of cases, if the hole in the dam be not too large.

For crown, lingual and labial cavities, if the teeth be ever so crowded, I never find it necessary to force the rubber between, but simply allow it to remain drawn down tightly over them.

I have used the above method with great satisfaction for the last year, and think it a valuable acquisition to my knowledge in practice. I do not think I should rate it at par with the *discovery* of the dam, but, joking aside, I do think the dam, clamps, and forceps, with the above method of application, should be inseparable.—*Dental Miscellany.*

